# SSVEP unsupervised adaptive feature recognition method based on self-similarity of same-frequency signals

**DOI:** 10.3389/fnins.2023.1161511

**Published:** 2023-08-01

**Authors:** Wenqiang Yan, Bo He, Jin Zhao

**Affiliations:** ^1^School of Mechanical Engineering, Xi’an Jiaotong University, Xi’an, China; ^2^State Key Laboratory for Manufacturing Systems Engineering, Xi’an Jiaotong University, Xi’an, China

**Keywords:** steady-state visual evoked potential, brain-computer interface, unsupervised adaptive classification algorithm, co-frequency self-similarity, signal detection

## Abstract

**Introduction:**

As an important human-computer interaction technology, steady-state visual evoked potential (SSVEP) plays a key role in the application of brain computer interface (BCI) systems by accurately decoding SSVEP signals. Currently, the majority SSVEP feature recognition methods use a static classifier. However, electroencephalogram (EEG) signals are non-stationary and time-varying. Hence, an adaptive classification method would be an alternative option to a static classifier for tracking the changes in EEG feature distribution, as its parameters can be re-estimated and updated with the input of new EEG data.

**Methods:**

In this study, an unsupervised adaptive classification algorithm is designed based on the self-similarity of same-frequency signals. The proposed classification algorithm saves the EEG data that has undergone feature recognition as a template signal in accordance with its estimated label, and the new testing signal is superimposed with the template signals at each stimulus frequency as the new test signals to be analyzed. With the continuous input of EEG data, the template signals are continuously updated.

**Results:**

By comparing the classification accuracy of the original testing signal and the testing signal superimposed with the template signals, this study demonstrates the effectiveness of using the self-similarity of same-frequency signals in the adaptive classification algorithm. The experimental results also show that the longer the SSVEP-BCI system is used, the better the responses of users on SSVEP are, and the more significantly the adaptive classification algorithm performs in terms of feature recognition. The testing results of two public datasets show that the adaptive classification algorithm outperforms the static classification method in terms of feature recognition.

**Discussion:**

The proposed adaptive classification algorithm can update the parameters with the input of new EEG data, which is of favorable impact for the accurate analysis of EEG data with time-varying characteristics.

## 1. Introduction

A brain-computer interface (BCI) can be defined as a system that transforms brain activity patterns into messages or commands for interactive applications (Anumanchipalli et al., 2019; Heelan et al., 2019; Lamti et al., 2019; Spiegel et al., 2019; Kubanek et al., 2020). Steady-state visual evoked potential (SSVEP)-based BCI is one of the most widely used scalp electroencephalogram (EEG) BCI systems. SSVEP is a steady-state EEG response recorded on the scalp at the same frequency as the stimulus frequency and its multiples by presenting the subjects with stimulus blocks flickering at a certain frequency (Wieser et al., 2016; Yan et al., 2018). In standard SSVEP-BCI systems, the user can view multiple concurrent stimuli located at various positions in the visual field (e.g., multiple light flicker patterns on the screen). Each stimulus is presented at a fixed frequency and represents a specific BCI output (e.g., outputting a specific letter or moving a wheelchair in a specific direction), and the user outputs a control command by directing their gaze at the stimulus representing the desired BCI output (Yan et al., 2021a,b, 2022).

SSVEP-BCI identifies the user’s gazed target by looking for certain frequency components in the EEG signals. Due to the significant frequency domain characteristics of SSVEP, most early studies on SSVEP-BCI used a power spectrum-based method to detect EEG signals, i.e., to identify the user’s gazed target by extracting the frequency points with peak energy in the frequency domain (Vialatte et al., 2010). However, this method is susceptible to noise interference and has limited performance. In light of this, the SSVEP’s time domain information has recently been incorporated into the analysis. Canonical correlation analysis (CCA) is a multivariate statistical method that measures the linear correlation between two groups of signals (Lin et al., 2007), which has been widely used in SSVEP-BCI. The goal of CCA is to find two linear combinations (denoted as spatial filters) that maximize the correlation coefficient between multichannel EEG signals and reference signals made up of sine and cosine signals. For the EEG data to be classified, the canonical correlation coefficients between the EEG and the reference signals at different frequencies are calculated, and the frequency corresponding to the maximum correlation coefficient is determined as the target frequency. With sine and cosine as reference signals, the CCA method does not fully utilize the intrinsic feature information of the EEG itself. In light of this, a template-based CCA method that has the same fundamental concept and calculation process as the conventional CCA method was proposed (Bin et al., 2011). The difference lies in that the template-based CCA method superimposes and averages previously recorded EEG data as reference signals to estimate the spatial filter. Compared with conventional CCA, template-based CCA employs the user’s intrinsic EEG signals as template signals, which can effectively reflect the user’s EEG specificity and exhibit improved performance. To fully utilize the fundamental frequency and harmonic information of SSVEP to further improve the recognition accuracy, a filter-bank CCA (FBCCA) method was proposed (Chen et al., 2015). In this method, filter-bank analysis is applied to filter the EEG data into different sub-bands, followed by CCA analysis on each sub-band to obtain the canonical correlation coefficients. Additionally, the obtained correlation coefficients of each frequency band are weighted, and their sum is used as the final feature discriminative coefficient. CCA and FBCCA are unsupervised (or training-free) methods, while template-based CCA is a supervised (or training-based) method due to the necessity of collecting user training data. In addition to template-based CCA methods, supervised methods such as extended canonical correlation analysis (eCCA) (Chen et al., 2015) and task-related component analysis (TRCA) (Nakanishi et al., 2018) were also proposed. Based on the CCA algorithm, eCCA identifies the user’s gaze target by solving the spatial filters between training, testing, and reference signals made up of sine and cosine functions, performing spatial filtering on these multichannel EEG signals, and then measuring the correlation between the spatially filtered signals. The main idea of TRCA is to find a linear combination that maximizes the repeatability of the event-related frequency components in the raw data over multiple trials, and this method has achieved good results in SSVEP target recognition.

The aforementioned algorithms do not reuse the EEG information that has undergone feature recognition and therefore, the classifier parameters are unchanged. Since EEG signals are non-stationary and time-varying (Mouraux and Iannetti, 2008), adaptive classification technology was developed to track the possible changes in EEG feature distribution and obtain improved classification results. There are three types of adaptive classification method, which are supervised (Shenoy et al., 2006; Schlögl et al., 2010), semi-supervised (Li et al., 2008; Li and Guan, 2008), and unsupervised (Blumberg et al., 2007; Vidaurre et al., 2011; Kindermans et al., 2014; Zanini et al., 2017). The supervised adaptive classification method trains the classification model using EEG data with known labels and requires that the newly input EEG data to have known labels. But in actual BCI systems, the labels of the newly input EEG data are determined by the trained classification model and thus, they are not necessarily true or accurate. Therefore, the supervised adaptive classification method is not suitable for BCI systems. The semi-supervised adaptive classification method uses both the initial labeled data and the newly input unlabeled data to adapt the classifier. In BCI, the semi-supervised adaptive classification algorithm first trains the classification model using the training data with known labels, then uses that model to estimate the label of the newly input unlabeled EEG data, and finally adapts/retrains the classifier using the estimated labeled data combined with the training data with known labels. The above process is repeated as a new batch of unlabeled EEG data becomes available. The unsupervised adaptive classification algorithm is utilized when the training data used to initially train the classification model have unknown labels, or even if there are no training data, and the labels of the newly input EEG data are also unknown. Adaptive classification algorithms have been explored for applications in BCI systems based on SSVEP, motor imagery (MI) and event-related potential (ERP). For SSVEP-BCI, Wong et al. (2022) proposed an online adaptation scheme to tune the spatial filters using the online unlabeled data from previous trails. For MI-BCI, Hsu (2011) proposed adaptive linear discriminant analysis based on Kalman filtering to track the distribution of each category. To deal with possibly imperfect labels in supervised adaptation, Yoon et al. (2009) proposed and evaluated offline an adaptive Bayesian classifier based on sequential Monte Carlo sampling that explicitly models uncertainty in the observed labels. For ERP-BCI, Woehrle et al. (2015) explored an adaptive support vector machine, adaptive linear discriminant analysis, adaptive linear classifiers based on stochastic gradient, and online passive-aggressive (PA) algorithms. The performance of adaptive classification algorithms has not been fully verified in BCI systems based on visual evoked potentials. In this study, an unsupervised adaptive algorithm framework for SSVEP classification is proposed.

Currently, FBCCA is the most commonly used unsupervised method for SSVEP classification. In this paper, an unsupervised adaptive classification algorithm (UAC) is designed based on FBCCA. Frequency information is the key feature of SSVEP signals. Apparently, signal-to-noise ratio (SNR) would increase with the superimposition of signals with the same frequency, while the superimposition of signals with varying frequencies would lead to a decrease in SNR. Based on this fundamental conclusion, batches of newly input EEG data are saved as template signals according to their estimated labels, and the template signals are continuously updated as the number of batches of the input EEG increases. In practical applications, the newly input EEG data are first superimposed with the saved template signals at different frequencies to obtain new testing signals. The newly input EEG data belong to one of the *n* stimulus frequencies. Their SNR is enhanced when they are superimposed with the template signals at the same frequency and weakened when superimposed at a different frequency. FBCCA is used for analyzing the testing signals produced by superimposing template signals, and the obtained correlation coefficient is used as the SSVEP feature recognition coefficient. Meanwhile, this study integrates the correlation coefficient obtained by the testing signal superimposed with the template signals and the correlation coefficient obtained by the fundamental FBCCA method as the final feature discrimination coefficient. The effectiveness of the proposed unsupervised adaptive classification algorithm compared with the static classification method in improving SSVEP recognition accuracy was verified on two public datasets.

## 2. Methods

### 2.1. Data

#### 2.1.1. Benchmark dataset

This dataset (Wang et al., 2017) includes SSVEP-BCI recordings of 35 healthy subjects focusing on 40 characters flickering at different frequencies (8–15.8 Hz with an interval of 0.2 Hz). For each subject, the experiment consisted of six blocks, where each block contained 40 trials corresponding to all 40 characters presented in a random order. The sampling frequency of the data is 250 Hz. The SSVEP signal analysis channels selected in this study were O1, O2, Oz, PO3, PO4, POz, PO5, and PO6.

#### 2.1.2. UCSD dataset

In the UCSD dataset (Nakanishi et al., 2015), there are 10 subjects’ SSVEP data collected from 15-run experiment. In each run, the subject was instructed to gaze at 12 visual stimuli one by one while his/her EEGs were recorded from eight electrodes (O1, O2, Oz, PO3, PO4, POz, PO5, and PO6) placed around parietal and occipital cortex. Twelve stimulus targets were tagged with different frequencies (*f*_0_ = 9.25 Hz, Δ*f* = 0.5 Hz) and phases (*ϕ*_0_ = 0, Δ*ϕ* = 0.5 π). At the beginning of each trial, a red square appeared for 1 s at the position of the target stimulus. After that, all stimuli started to flicker simultaneously for 4 s on the monitor. The sampling frequency of the data is 256 Hz.

### 2.2. Canonical correlation analysis

For EEG data *X* recorded from multiple channels and the reference signal *Y*, the goal of CCA is to find two projection vectors *w_X_* and *w_Y_* so that the two groups of linear combination signals *w_X_*^T^*X* and *w_Y_*^T^*Y* have the largest correlation coefficients. The canonical correlation coefficient was calculated by:


(1) $$\rho =\max\frac{E\left(w_{X}^{T}XY^{T}w_{Y}\right)}{\sqrt{E\left(w_{X}^{T}XX^{T}w_{X}\right)E\left(w_{Y}^{T}YY^{T}w_{Y}\right)}}$$

The reference signals *Y* were constructed at the stimulation frequency *f*:


(2) $$Y_{f}=\left\{\begin{array}{c} \cos2\pi ft\\ \begin{array}{c} \sin2\pi ft\\ \begin{array}{c} \ldots \\ \begin{array}{c} \cos2k\pi \mathrm{ft}\\ \sin2k\pi \mathrm{ft} \end{array} \end{array} \end{array} \end{array}\right\},t=1/f_{s},\ldots ,N_{s}/f_{s}$$

where *k* is the number of harmonics, *f_s_* is the sampling rate, and *Ns* represents the number of sample points. By calculating the canonical correlation coefficients between *X* and the reference signals at all stimulus frequencies, and the corresponding target with the maximum correlation coefficient is identified as the user’s focused target.

### 2.3. Filter bank canonical correlation analysis

In FBCCA method, *D* sub-band filters with different upper and lower cutoff frequencies filter the EEG signal *X* to obtain sub-band filtered signals *X*_*SB,*1_, *X*_*SB,*2_,…, *X_SB,D_*. Then, the maximum canonical correlation coefficient of each sub-band signal at frequency *f* is calculated by CCA:


(3) $$\rho_{SB,d}=\max\frac{w_{X}^{T}X_{SB,d}Y_{f}{}^{T}w_{Y}}{\sqrt{\left(w_{X}^{T}X_{SB,d}X_{SB,d}^{T}w_{X}\right)\;\left(w_{Y}^{T}Y_{f}Y_{f}^{T}w_{Y}\right)}}$$

where *X_SB,d_* represents the *d-*th sub-band signal. FBCCA integrates the maximum canonical correlation coefficient of *D* sub-band signals according to the weighting function as the feature discriminant coefficient at the frequency *f*:


(4) $${\tilde{\rho }}_{f}=\overset{D}{\underset{d=1}{\sum }}w\left(d\right)\;•\;{\left(\rho_{SB,d}\right)}^{2}$$

where *w*(*d*) represents the weight corresponding to the canonical correlation coefficient of the *d*-th sub-band signal:


(5) $$w\left(d\right)=d^{-a}+b,d\in \left[1\;D\right]$$

where *a* = 1.25 and *b* = 0.25 (Chen et al., 2015). After calculating the integration coefficients at all stimulation frequencies, FBCCA determines the frequency corresponding to the maximum coefficient as the gaze target frequency.

### 2.4. SSVEP unsupervised adaptive classification algorithm based on self-similarity of same-frequency signals

**Figure 1:**
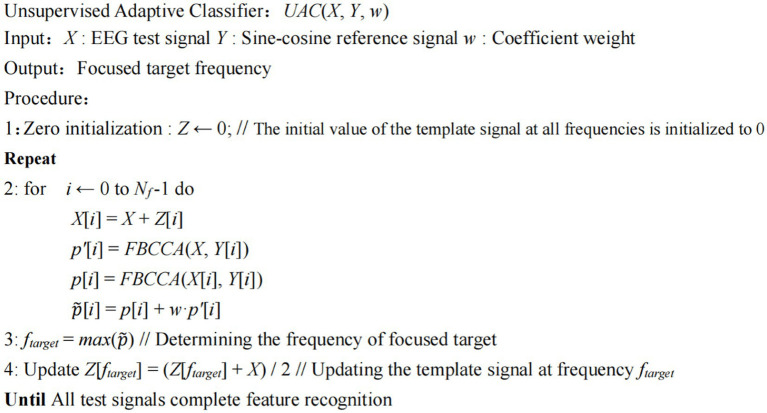
The algorithm process of the proposed unsupervised adaptive classifier.


The algorithm steps of the proposed unsupervised adaptive classification method (UAC) are shown in Figure 1. First, the template signal *Z*∈*O^Nt × Nc×Nf^* is initialized to zero, where *N_t_* represents the number of sampling points, *N_c_* represents the number of electrode channels, and *N_f_* represents the number of stimulus targets. Given that the frequency of testing signal *X*∈R*^Nt × Nc^* belongs to one of *N_f_* stimulus frequencies, when the signal is superimposed with a template signal of the same frequency, SNR would be enhanced. Contrarily, when the signal is superimposed with a template signal of a different frequency, SNR would be weakened. Therefore, based on the self-similarity of same-frequency signals, this study superimposed testing signal *X* with template signals of different frequencies respectively, to obtain a new set of testing signals [*X*_1_, *X*_2_, …, *X_Nf_*]:

(6) $$X_{i}=X+Z_{i}$$

where *Z_i_*∈R*^Nt × Nc^* denotes the template signal at stimulus frequency *f_i_* in *Z*. Then, FBCCA was used for analyzing the newly obtained testing signals [*X*_1_, *X*_2_,…, *X_Nf_*] and the reference signals of the corresponding frequency, respectively, to obtain the correlation coefficients [*p*_1_, *p*_2_,…, *p_Nf_*]:


(7) $$p_{i}=\mathrm{FBCCA}\;\left(X_{i},Y_{i}\right)$$

where *X_i_* denotes the superimposed result of testing signal *X* and the template signal at frequency *f_i_* in *Z*, and *Y_i_* denotes the reference signal at frequency *f_i_*. In the basic FBCCA method, the correlation coefficient obtained from testing signal *X* and the reference signal is used as the feature discrimination coefficient:


(8) $$p_{i}^{\prime }=\mathrm{FBCCA}\;\left(X,Y_{i}\right)$$

Characteristic coefficient integration is performed in the proposed unsupervised adaptive algorithm framework, i.e., feature coefficients *p_i_* and *p_i_*’ are integrated as the final feature discrimination coefficient:


(9) $${\tilde{p}}_{i}=p_{i}^{\prime }+w•p_{i}$$

where *w* is the weight of the coefficient *p_i_*. After the integrated coefficients are calculated at all frequencies, the frequency *f*_max_ corresponding to the maximum coefficient is determined as the target frequency. Meanwhile, the template signal is updated by adding the testing signal *X* to the template signal at frequency *f*_max_. Assuming that the raw signal at frequency *f*_max_ in template signal *Z* is *Z_f_*, then the updated template signal is:


(10) $$Z_{f}^{\prime }=\frac{X+Z_{f}}{2}$$

The updated template signal is used as the new template signal for feature recognition of the next batch of input EEG signals. In the basic FBCCA method, the features information of testing signal *X* are not reused once recognized, because the recognition process for the subsequent batch of input EEG signals starts. In the proposed adaptive classification algorithm, the EEG data for feature recognition that have been completed are saved as template signals according to their estimated labels, and the template signals are updated with the input of new batches of EEG data. As a result, the possible changes in EEG feature distribution can be tracked.

## 3. Results

### 3.1. Self-similarity of same-frequency signals application in SSVEP feature recognition

Frequency is the main information used to identify the features of SSVEP signals. Because the SSVEP signal is time-locked and phase-locked (Chen et al., 2015), the superposition average of same frequency signals can enhance the SNR, while the superposition average of different frequency SSVEP signals can weaken the SNR. The EEG data which have undergone feature recognition are saved as template signals according to their estimated labels, and the newly input testing signal is superimposed with the template signals at different frequencies. Only when the testing signal has the same frequency as the template signal can the frequency component of the testing signal be enhanced. Therefore, signal features obtained by the superimposition of the testing and template signals can be extracted to identify the user’s gaze target.

**Figure 2:**
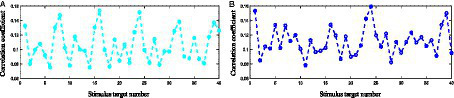
**(A)** The classification results obtained by CCA analysis of the original testing signal. **(B)** The classification results obtained by performing CCA analysis following the superimposition process of the testing signal with the template signals.

The *Benchmark* dataset is used for analysis, and the classification results of EEG signals when Subject 22 gazes at the 24th stimulus target (at a stimulus frequency of 15.4 Hz) are shown in Figure 2, where the horizontal axis represents the number of the stimulus target and the vertical axis represents the correlation coefficients calculated from the testing signal and the reference signals of different frequencies. Herein, the time length of the data to be analyzed is 1 s and the number of harmonics of the reference signals is set to 5. As can be seen, the correlation coefficients have larger values at 15 Hz, 15.2 Hz, 15.4 Hz, and 15.6 Hz, which means that the misrecognition of the gaze target is most likely to occur at frequencies close to the real stimulus frequency. In Figure 2, the largest correlation coefficient appears at 15.2 Hz, so the BCI system would misrecognize that the user is gazing at the 16th stimulus target. Assuming that the data at 40 frequencies in the second block of Subject 22 are the constructed template signals, Figure 2B shows the classification results obtained by performing CCA analysis following the superimposition process of the testing signal with the template signals. As observed, the correlation coefficients at 15 Hz, 15.2 Hz, and 15.6 Hz are suppressed, while the correlation coefficients at the real stimulus frequency of 15.4 Hz are highlighted, thereby accurately identifying the user’s gaze target. Since the real frequency of the testing signal is 15.4 Hz, superimposing this signal with the template signal at the same frequency would significantly enhance the 15.4 Hz frequency component of the testing signal, resulting in a higher correlation coefficient when CCA is performed. In summary, the application of self-similarity of same-frequency signals in the proposed adaptive classification algorithm has a favorable impact for the accurate recognition of SSVEP features.

### 3.2. Necessity of feature coefficients integration

**Figure 3:**
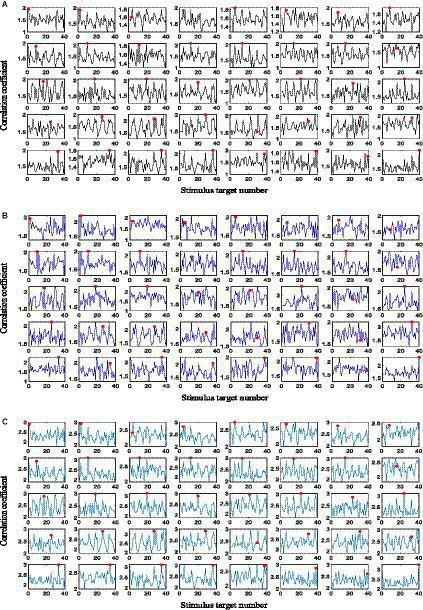
**(A)** The classification results obtained by the basic FBCCA method. **(B)** The classification results obtained by performing FBCCA analysis following the superimposition process of the testing signal with the template signals. **(C)** The classification results obtained by integrating feature discriminant coefficients.

Taking the experimental data of the 6th block of Subject 6 in the *Benchmark* dataset as an example, this section demonstrates the necessity of integrating the feature coefficient obtained by the basic FBCCA method and the one obtained by superimposing the testing signal with the template signals before performing FBCCA. The time length of the data to be analyzed is 1 s, and the number of filter banks and harmonics of the reference signals in the FBCCA method are set to 5. Classification results obtained by the basic FBCCA method when the user gazes at 40 stimulus targets, respectively, are shown in Figure 3, where the horizontal axis of each subplot represents the stimulus target number, the vertical axis represents the feature coefficient obtained by FBCCA, and the red dots mark the corresponding feature coefficients of the real gaze targets. Figure 3B shows the classification results obtained by superimposing the testing signal with the template signals before performing FBCCA. Herein, the template signals are constructed from the first 5 blocks of data of Subject 6 according to the method proposed in Section 2.4. In Figure 3, the recognition accuracy obtained by using the basic FBCCA method is 0.575, whereas the recognition accuracy obtained by superimposing the testing signal with the template signals and then performing FBCCA is 0.4 as shown in Figure 3B. As the proposed adaptive classification algorithm constructs template signals based on the estimated label of the newly input EEG data, due to the possible misrecognition of the estimated label, the constructed template signals might not be accurate. Meanwhile, the initialization value of the template signals at all frequencies is 0. The template signals gradually have a real value with the input of new batches of EEG data, which means that the template signals at some frequencies might be in an empty state during the SSVEP feature recognition. As a result, the recognition accuracy obtained by superimposing the testing signal and the template signals and then performing FBCCA analysis is lower than that obtained by the basic FBCCA method. Notably, the classification results shown in Figures 3A,B are complementary. For example, gaze targets 11, 25, 32, and 37 are misrecognized in Figure 3 but correctly recognized in Figure 3B, and gaze targets 5, 7, 8, and 12 are misrecognized in Figure 3B but correctly recognized in Figure 3. Since the input data are different, the two corresponding feature discriminant coefficients of Figures 3A,B have different classification preferences, making it possible to integrate the two into a strong classifier.

**Figure 4:**
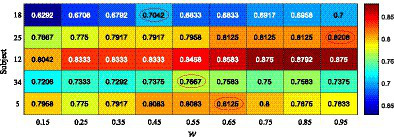
The recognition accuracies of five selected subjects under different weights of ensemble coefficient.

The feature coefficients in Figures 3A,B are integrated according to Equation (9), and five subjects are randomly selected from the *Benchmark* dataset to determine the value of the weight coefficient *w*. Figure 4 shows the recognition accuracy of the five subjects under different values of *w*, and the time length of the data to be analyzed is 1 s. As observed, the optimized value of weight *w* varies greatly among subjects. In this study, according to the averaged accuracy, the value of *w* is determined as 0.45. Figure 3C shows the classification results obtained by integrating two feature discriminant coefficients. As can be seen, gaze targets 1 and 31 are misrecognized in both Figures 3A,B but correctly recognized in Figure 3C. The recognition accuracy of Figure 3C is 0.675, which is higher than the recognition accuracy obtained by using the corresponding discriminant coefficients of Figures 3A,B alone. By using the same method, the weight coefficient of Equation (9) in the *UCSD* dataset is determined as 0.65. These experimental results demonstrate the necessity and effectiveness of integrating feature discriminant coefficients in the proposed adaptive classification method.

### 3.3. Factors influencing feature recognition performance of the proposed adaptive classification method

**Figure 5:**
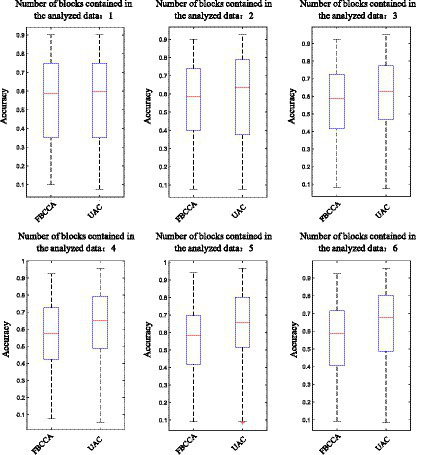
The recognition accuracies of the basic FBCCA method and the unsupervised adaptive classification method under 1–6 blocks of data.

The template signals of the proposed adaptive classification algorithm are continuously updated with the input of new batches of EEG data, and the initial empty templates at each frequency will be gradually filled with real values. Meanwhile, the template signals become increasingly accurate as more EEG data are superimposed over the template signals. Therefore, the feature recognition performance of the adaptive classification method will progressively improve with the continuous use of the BCI system. To verify this conclusion, the data in the *Benchmark* dataset are used for analysis. The recognition accuracies of the basic FBCCA method and the proposed unsupervised adaptive classification method (UAC) under 1–6 blocks of data are calculated respectively, and the experimental results are shown in Figure 5. Herein, the time length of the data to be analyzed is 1 s, and the number of filter banks and reference signals harmonics in the FBCCA method are set to 5. The accuracy calculation begins by averaging the accuracies of all blocks for each subject, and the resulting average recognition accuracies for all subjects are then averaged as the final result. The results in Figure 5 show that when the number of blocks contained in the analyzed data is 1–6, the adaptive classification method can improve the recognition accuracy of the basic FBCCA method by 1.25, 5, 4.17, 7.5, 7.25, and 8.96%, respectively, and the increase in feature recognition performance is proportional to the amount of data of the BCI system. Therefore, with the continuous use of the BCI system, the template signals constructed by the adaptive classification method will become more accurate and the feature recognition performance will gradually improve.

**Figure 6:**
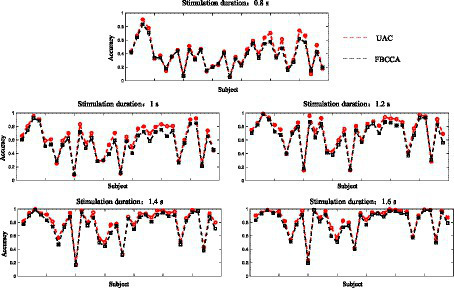
The recognition accuracies obtained by the basic FBCCA method and the adaptive classification method for each subject at stimulus durations of 0.8 s, 1 s, 1.2 s, 1.4 s, and 1.6 s.

The proposed adaptive classification method adds the newly input EEG data to the template signals at the corresponding frequency according to their estimated labels. The estimated labels of subjects with a good SSVEP response are more accurate (i.e., higher recognition accuracy), making the constructed template signals are also more accurate. Hence, the adaptive classification method is more effective in improving the feature recognition performance of subjects with a good SSVEP response. The recognition accuracies obtained by the basic FBCCA method and the adaptive classification method for each subject at stimulus durations of 0.8 s, 1 s, 1.2 s, 1.4 s, and 1.6 s are shown in Figure 6, where the horizontal axis represents the subject number, and the vertical axis represents the recognition accuracy. As observed, under all stimulus durations, the adaptive classification method greatly improves the feature recognition performance for subjects with a good SSVEP response, including subjects 3, 4, 26, 28, 31, and 32, but exhibits a small performance improvement for subjects with a limited SSVEP response. This can be attributed to the fact that subjects with limited SSVEP responses have relatively low recognition accuracy, more incorrectly estimated labels, and poor accuracy of the constructed template signals, resulting in the mediocre performance improvement of the adaptive classification method.

In summary, the longer the BCI system is used, the more data can be exploited to construct template signals, and the better the feature recognition performance of the adaptive classification method. Meanwhile, good SSVEP responses lead to accurate estimated labels, accurate constructed template signals, and an effective adaptive classification method in improving feature recognition performance.

### 3.4. Feature recognition performance of adaptive and static classification methods

**Figure 7:**
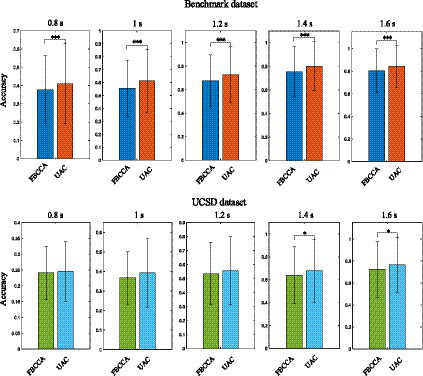
The SSVEP feature recognition performance of the FBCCA method with that of the unsupervised adaptive classification method using the Benchmark and UCSD dataset. The symbol ‘*’, ‘**’ and ‘***’ represent significance test value *p* < 0.05, *p* < 0.001 and *p* < 0.0001, respectively.

**Figure 8:**
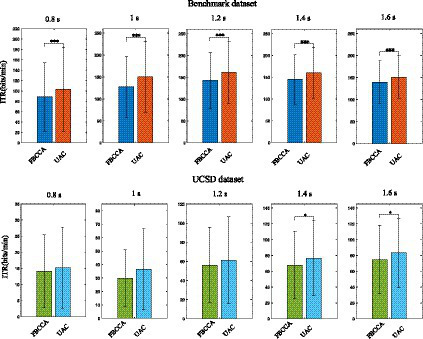
The ITRs of the FBCCA method with that of the unsupervised adaptive classification method using the Benchmark and UCSD dataset. The symbol ‘*’, ‘**’ and ‘***’ represent significance test value *p* < 0.05, *p* < 0.001 and *p* < 0.0001, respectively.

For static classification method (e.g., FBCCA-based), after the features of the input EEG data are recognized, the feature recognition process of the next batch of input data starts, and the recognized EEG data information will not be reused. For the proposed adaptive classification method, the EEG data which have undergone feature recognition are saved as template signals according to their estimated labels. As new input EEG data are obtained, the template signals would be re-estimated and updated, which enables the adaptive classification method to track possible changes in the EEG feature distribution. Figure 7 compares the SSVEP feature recognition performance of the static classification method with that of the unsupervised adaptive classification method (UAC) using the *Benchmark* and *UCSD* dataset. In accuracy computation, the average recognition accuracy of each subject was obtained by first averaging the accuracies of 6 blocks for each subject, and these average recognition accuracies obtained for 34 subjects were then averaged to obtain the final analysis result. Paired wilcoxon signed-rank test was used to determine significant differences (defined as *p* < 0.05) in accuracy for different methods. For the *Benchmark* dataset, the number of filter banks and reference signal harmonics in the FBCCA method were both set to 5, and the weight coefficient in Equation (9) was set to 0.45. For the *UCSD* dataset, the number of filter banks and reference signal harmonics in the FBCCA method were both set to 3, and the weight coefficient in Equation (9) was set to 0.65. Experimental results in Figures 7, 8 show that, for the *Benchmark* dataset, when the stimulus duration was 0.8 s, 1 s, 1.2 s, 1.4 s, and 1.6 s, the adaptive classification method could improve the recognition accuracy of FBCCA by 3.36, 5.96, 5.18, 4.84, and 4.02%, and could improve the information transmission rate (ITR) (Oikonomou et al., 2019) of FBCCA by 14.7, 23.1, 18.8, 15.4, and 11.5 bits/min, respectively; for the *UCSD* dataset, when the stimulus duration was 0.8 s, 1 s, 1.2 s, 1.4 s, and 1.6 s, the adaptive classification method could improve the recognition accuracy of FBCCA by 0.45, 2.46, 2.11, 3.56, and 4.28%, and could improve the ITR of FBCCA by 1.05, 6.56, 5.39, 8.8, and 8.85 bits/min, respectively. Paired wilcoxon signed-rank test shows that there is a significant difference between the accuracies and ITRs obtained by the adaptive and static classification methods (*p < 0.05, **p < 0.001, ***p < 0.0001). The tests on public datasets suggested that the adaptive classification method is more suitable for the SSVEP-BCI system.

The feature recognition performance of the adaptive classification method proposed in this study with the method proposed in Wong et al. (2022) was compared. The OACCA method proposed in Wong et al. (2022) uses the same public dataset as the method proposed in our study. The comparison results show that both the OACAA method and the adaptive method proposed in our study effectively improve the recognition accuracy of the Benchmark dataset. The performance of the OACCA method is better than the adaptive classification method proposed in our study. Considering that OACCA method and the method proposed in our paper are based on different technical routes, both of them have reference significance for the design of adaptive classification methods. Therefore, although the proposed method does not achieve better performance than the OACCA method, it provides a new idea of adaptive classification method design.

## 4. Discussion

SSVEP-BCI is an important human-computer interaction technique, and the accurate decoding of SSVEP signals is the key to ensuring the widespread application of SSVEP-BCI systems. Common SSVEP classification techniques include the matrix classifier (spatial filter) (Wong et al., 2020; Oikonomou, 2022), Riemannian geometry classifier (Kalunga et al., 2016), tensor classifier, transfer learning, and deep learning, all of which are static classifiers, i.e., the classifier parameters are fixed. However, EEG signals are non-stationary and time-varying. Since adaptive classification method parameters can be re-estimated and updated as new EEG data are obtained, adaptive classification methods are more suitable for EEG signal decoding. Adaptive classification methods can be divided into three categories, which are supervised, semi-supervised, and unsupervised. The unsupervised adaptive classification method is the most challenging to design due to the lack of training data and the unknown labels of the newly input EEG data. Therefore, based on the self-similarity of same-frequency signals, an unsupervised adaptive classification method for visual evoked potential classification was proposed in this study.

In the proposed adaptive classification method, the EEG data which have undergone feature recognition were saved as template signals according to their estimated labels, and the newly input testing signal was superimposed with the template signals at each frequency to obtain the new signals to be analyzed. Only when the testing and template signals have the same frequency can the frequency component of the testing signal be enhanced. The classification results obtained by raw testing signals and the superimposition of a testing signal with template signals were compared. The results demonstrated that the analysis by FBCCA after the superimposition of the testing signal with the template signals could highlight the feature coefficients at the real stimulus frequencies and suppress the feature coefficients at the non-gaze target frequencies. Meanwhile, given that the adaptive classification method constructed the template signals based on the estimated labels of the EEG data, the constructed template signals might not be completely accurate due to the possible misidentification of the estimated labels. Additionally, with zero initialization, template signals at all frequencies gradually acquire a real value with the input of new batches of EEG data, which means that template signals at some frequencies may be in an empty state during the SSVEP feature recognition. As a result, the recognition accuracy obtained by superimposing the testing and template signals before performing FBCCA analysis was lower than that obtained by the basic FBCCA method. Notably, the classification results obtained by superimposing the testing signal to the template signals are complemented by the classification results obtained by using the testing signals directly. Since the two classification methods have different classification preferences, the proposed adaptive classification method integrated the feature coefficients of the two methods to obtain improved classification performance.

The proposed adaptive classification method is an unsupervised method in which the initial value template signals at each frequency is zero and the template signals are progressively filled with real values as more EEG data are input. Meanwhile, as the feature recognition process proceeds, more EEG data would be superimposed and averaged with the template signals, which makes the template signals increasingly accurate with the persistent use of the BCI system. By analyzing the recognition accuracies with 1–6 blocks of data input to the BCI system, it was found that the improvement in feature recognition performance of the adaptive classification method over the basic FBCCA method was proportional to the amount of data input to the BCI system. Meanwhile, the estimated labels obtained from subject data with a good SSVEP response were more accurate, resulting in more accurate constructed template signals. Therefore, the adaptive classification method is more effective in improving the feature recognition performance of subjects with a good SSVEP response compared to those with a poor SSVEP response. To verify the feature recognition performance of the proposed unsupervised adaptive classification method, its recognition accuracy was compared with that of the static classification method (FBCCA) on two public datasets, and the experimental results showed that the adaptive classification method could achieve improved classification results for any stimulus duration. Therefore, an adaptive classification method is an alternative choice for SSVEP classification.

### 4.1. Future work

The SSVEP-based supervised (or training-based) feature recognition method usually requires collecting user training data in advance, which is a lengthy and time-consuming process. The unsupervised system can be transformed into a semi-supervised or even a supervised system without collecting user training data. The process is to save the new data input to the BCI system as training data, then apply the training feature recognition method (e.g., eCCA or TRCA) only to some frequencies where there are real-valued training data, and finally apply the non-trained feature recognition method to remaining frequencies. With the continuous input of EEG data, when the real-valued training data is available at all frequencies, the training feature method can be applied to all frequencies. By gathering user training data online, the new system can bridge the gap between supervised and unsupervised methods, avoiding the long data collection process and taking advantage of the high accuracy of training-based feature recognition methods. In this study, an unsupervised adaptive method was proposed. In the future, more studies can be conducted to explore the semi-supervised adaptive feature recognition algorithm framework and to investigate the adaptive method of classifier parameters in the presence of training data.

## 5. Conclusion

Given that EEG signals are non-stationary and time-varying, adaptive classification algorithm would be an alternative method for SSVEP feature classification than static classification method in tracking potential changes in EEG feature distribution, as their parameters can be re-estimated and updated with the input of new EEG data. In this study, the EEG data which have undergone feature recognition were saved as template signals according to their estimated labels, and then based on the self-similarity of same-frequency signals, the testing signal was superimposed with the template signals to produce the new signals to be analyzed. Experimental results demonstrated the feasibility and necessity of applying the self-similarity of same-frequency signals and integrating feature coefficients in the proposed adaptive classification method. Meanwhile, it is proved that the longer the BCI system is used and the better the SSVEP response of the user, the better the feature recognition performance of the adaptive classification method. By comparing the recognition accuracy of the adaptive and static classification methods on public datasets, it was demonstrated that the adaptive classification method is a more effective SSVEP feature recognition method. In the future, attention will be paid to the exploration of semi-supervised adaptive classification methods and the integration of unsupervised SSVEP-BCI systems with the training-based feature recognition methods.

## Data availability statement

The datasets presented in this study can be found in online repositories. The names of the repository/repositories and accession number(s) can be found at: (1) Benchmark Dataset: http://bci.med.tsinghua.edu.cn/download.html (2) UCSD Dataset: ftp://sccn.ucsd.edu/pub/cca_ssvep.

## Ethics statement

The studies involving human participants were reviewed and approved by (1) The Research Ethics Committee of Tsinghua University (2) The Human Research Protections Program of the University of California San Diego. The patients/participants provided their written informed consent to participate in this study.

## Author contributions

WY performed the data analyses and wrote the manuscript. BH performed the experiment. JZ contributed significantly to analysis and manuscript preparation. All authors contributed to the article and approved the submitted version.

## Funding

This research was supported by National Natural Science Foundation of China (NSFC) (no. 52105308), Fundamental Research Funds for the Central Universities (no. xhj032021010-02), and Scientific and Technological Innovation 2030 Key Project of Ministry of Science and Technology of China under grant (no. 2022ZD0209800).

## Conflict of interest

The authors declare that the research was conducted in the absence of any commercial or financial relationships that could be construed as a potential conflict of interest.

## Publisher’s note

All claims expressed in this article are solely those of the authors and do not necessarily represent those of their affiliated organizations, or those of the publisher, the editors and the reviewers. Any product that may be evaluated in this article, or claim that may be made by its manufacturer, is not guaranteed or endorsed by the publisher.
